# A Case Report of Riedel’s Thyroiditis Behaving as Anaplastic Thyroid Carcinoma: A Rare Presentation

**DOI:** 10.7759/cureus.95502

**Published:** 2025-10-27

**Authors:** Ahmed Lamey, Sherif Farahat, Salma Elsayed, Mark Magdy

**Affiliations:** 1 Department of General Surgery, Faculty of Medicine, Kafr Elsheikh University, Kafr Elsheikh, EGY; 2 Department of General Surgery, Burjeel Hospital, Al Ain, ARE; 3 Department of General Surgery, Kafr Elsheikh University Hospitals, Kafr Elsheikh, EGY; 4 Department of Colorectal Surgery, Hywel Dda University Health Board, Carmarthenshire, GBR

**Keywords:** anaplastic thyroid carcinoma, fibrosis, lymph-plasmacytic infiltrate, riedel’s thyroiditis, thyroid mass

## Abstract

This case report describes a 47-year-old male with a history of hypothyroidism, presenting with a rapidly enlarging neck mass, dysphagia, and dyspnea, raising clinical suspicion for anaplastic thyroid carcinoma (ATC). Initial investigations, including post-contrast CT and fine-needle aspiration cytology (FNAC), were inconclusive but suggested a possible malignant etiology. A total thyroidectomy was planned to relieve airway compression and rule out malignancy. Intraoperative findings revealed diffuse fibrosis and infiltration into surrounding structures, limiting surgical resection. Only a localized isthmectomy was performed.

Histopathological examination with further immunohistochemical staining (IHS) confirmed the diagnosis of Riedel’s thyroiditis (RT), a rare condition that mimicked ATC both clinically and radiologically. Postoperatively, the patient was treated with high-dose corticosteroids followed by tamoxifen, leading to significant clinical improvement and near-complete resolution of the thyroid mass within one year. This case underscores the importance of considering RT in the differential diagnosis of rapidly enlarging thyroid masses to avoid unnecessary, aggressive treatment.

## Introduction

Riedel’s thyroiditis (RT) is an uncommon form of chronic thyroiditis, first described by Bernhard Riedel in 1896 [1]. It accounts for less than 0.05% of all thyroid diseases and is often associated with other fibrosclerotic conditions. It is believed to represent either an autoimmune fibroinflammatory process or a localized manifestation of immunoglobulin G subclass 4 (IgG4)-related systemic disease, characterized by extensive fibrosis replacing normal thyroid parenchyma and extending into adjacent tissues [2]. Anaplastic thyroid carcinoma (ATC), on the other hand, is an aggressive and lethal form of thyroid cancer [3]. Both conditions can present with a rapidly enlarging neck mass, hoarseness, dysphagia, and symptoms of airway compression, making clinical differentiation challenging [4]. This report discusses a case of RT that clinically and radiologically mimicked ATC, leading to an initial misdiagnosis. The case underscores the importance of considering Riedel’s thyroiditis in the differential diagnosis of rapidly enlarging thyroid masses to avoid unnecessary aggressive treatment and the critical role of histopathological and immunohistochemical differentiation in establishing a definitive diagnosis and guiding appropriate management.

## Case presentation

A 47-year-old male with hypothyroidism was referred from the endocrine clinic for a long-standing neck swelling of more than 1 year, with a notable and relatively rapid increase in size. The neck circumference in serial examinations increased from approximately 34 cm to 40 cm over a 4-month period. The main symptom associated was the feeling of a strangulated neck. It was associated with dysphagia, dyspnea, and dyspepsia, even stridor, especially when lying down, but there was no hoarseness.

The general examination for IgG4-related disease was negative. There was no abdominal pain (suggestive of autoimmune pancreatitis), no jaundice (suggestive of sclerosing cholangitis), no back pain (suggestive of retroperitoneal fibrosis), no hematuria/proteinuria (suggestive of autoimmune renal disease), and no respiratory symptoms (suggestive of autoimmune pulmonary disease). 

**Figure 1:**
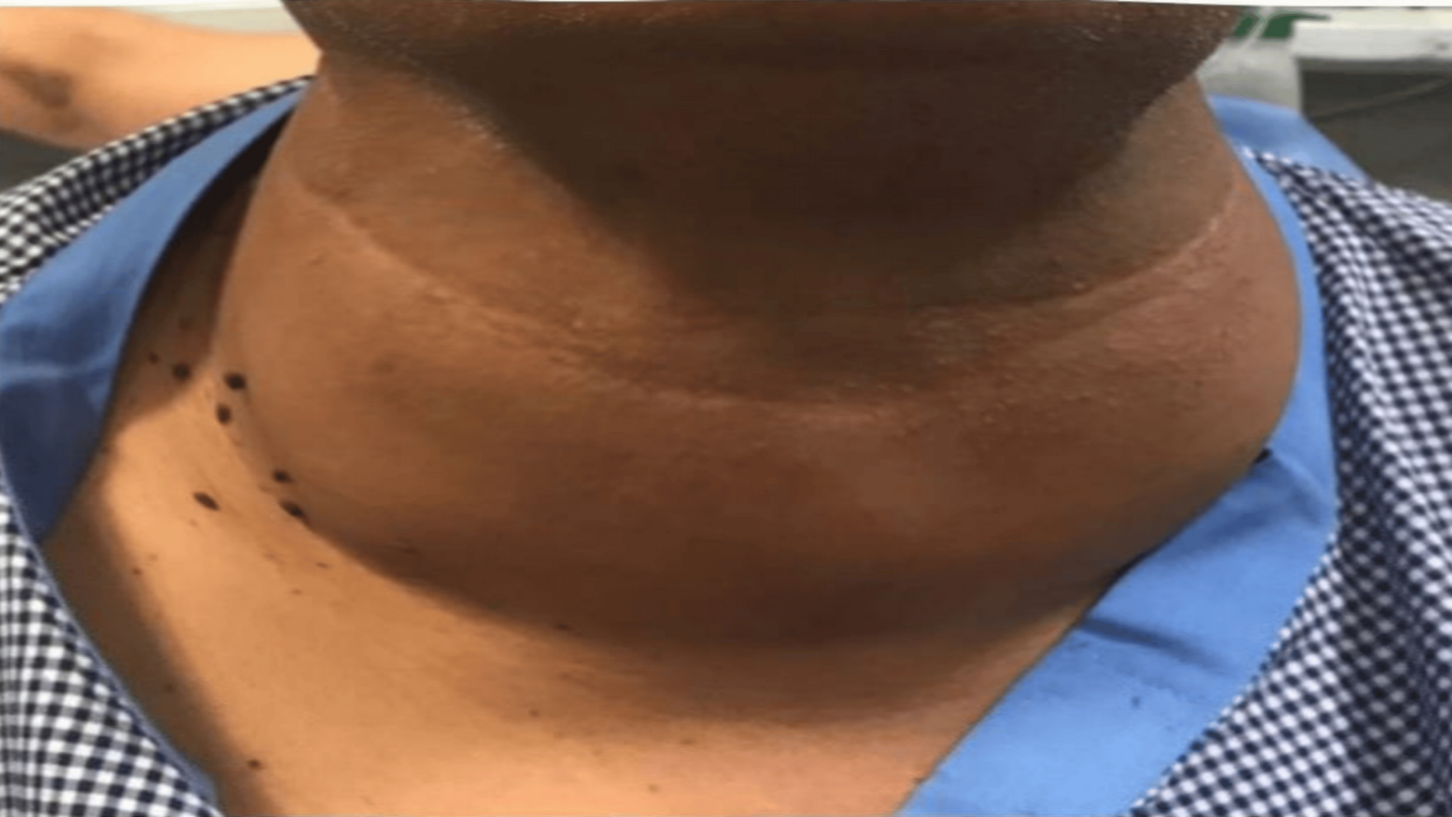
Inspection of the neck before surgery

On inspection of the neck, there were no definite inflammatory symptoms like pain, redness, hotness, fever, or malaise. There was an extremely large, hard swelling in the whole neck, moving up and down with difficulty, with deglutition associated with dilated neck veins (Figure 1).

On palpation, the swelling was very hard in consistency, involving the whole thyroid gland and isthmus. Although the upper border was palpable, the lower border was not, as the mass extended retrosternally, especially on the left side. There were no palpable lymph nodes in the neck or any other part of the body. Thyroid function tests (TFTs) showed hypothyroidism 4 months before referral (thyroid-stimulating hormone (TSH): 8.6 mIU/L, free thyroxine (T4): 9.4 pmol/L, free triiodothyronine (T3): 3.1 pmol/L), which was corrected by L-thyroxine treatment (150 µg/day). Preoperative TFTs were corrected to become TSH: 2.1 mIU/L, free T4: 15.0 pmol/L, and free T3: 4.4 pmol/L.

Initial neck ultrasound demonstrated a diffusely enlarged thyroid gland measuring 20×12×12 cm with marked hypoechogenicity, heterogeneous texture, ill-defined margins, and posterior acoustic shadowing compatible with dense fibrosis, with poor delineation of both internal jugular veins (infiltrated) and dilated posterior collaterals. Intra-thyroidal vascularity was reduced on color Doppler. No pathological cervical lymph nodes were identified. The mass is mainly wrapped around the trachea and extends retrosternally, especially on the left side.

**Figure 2:**
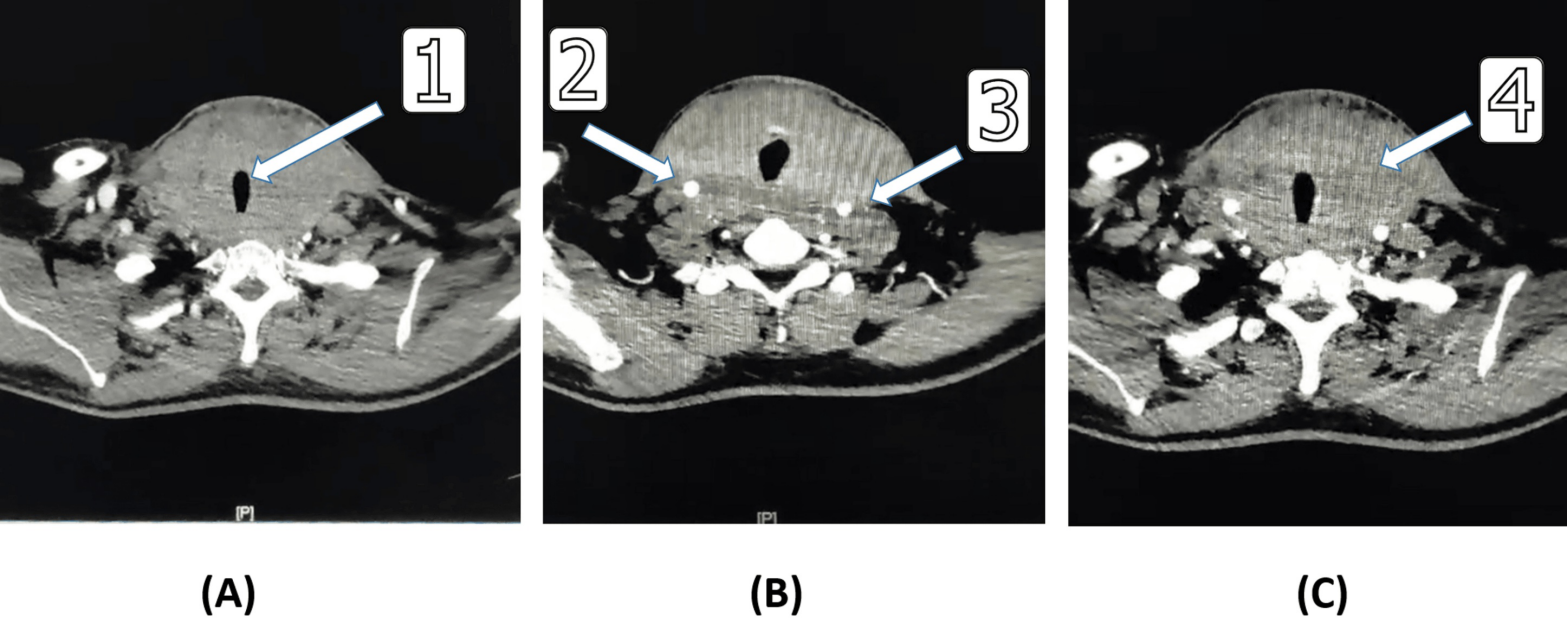
Post-contrast CT neck Arrow 1: Severe tracheal compression resulting in tracheomalacia. Arrow 2: Complete encasement of the carotid sheath, involving both the carotid artery and internal jugular vein. Arrow 3: Significant posterior compression of the esophagus, leading to dysphagia. Arrow 4: Marked retrosternal extension of the thyroid gland, more pronounced on the left side.

Post-contrast computed tomography (CT) neck showed a markedly enlarged single mass involving the whole thyroid gland and isthmus, extending to the hyoid bone above, markedly compressing the trachea and thyroid cartilage with no focal lesions, and extending retrosternally below. Post-contrast CT imaging also demonstrated that the thyroid mass was partially encasing both internal carotid arteries and internal jugular veins on both sides. Despite their outer wall compression, there was no evidence of tracheal invasion or carotid sheath invasion. There was calcified cricoid cartilage as a result of marked tracheomalacia associated with left retrosternal extension, with a few bilateral small cervical lymph nodes. CT suggested it could be thyroid lymphoma, anaplastic carcinoma, thyroiditis, or others (Figure 2).

Fine-needle aspiration cytology (FNAC) showed only plasma-lymphocytic infiltrates in the specimen; however, malignancy, such as ATC, could not be ruled out (Bethesda III). Repeating FNAC, the results remained indeterminate (Bethesda III). Although core needle biopsy (CNB) and serum IgG4 concentration can improve the diagnostic yield, they were deferred due to the urgent airway risks. Given the worsening compressive symptoms with stridor and the marked tracheal narrowing, we proceeded with surgical exploration to secure the airway and exclude malignancy.

Preoperative laboratory tests, including a complete blood count, liver enzymes, renal function, coagulation profile, serum thyroglobulin, and urinalysis, were all within the reference ranges. The erythrocyte sedimentation rate (ESR) was high at 200 mm/h. Preoperative abdominal ultrasound showed no pancreatic or hepatobiliary abnormalities. Preoperative indirect laryngoscopy showed no affection of either vocal cord.

A total thyroidectomy was planned to relieve stridor and tracheal compression and to rule out other malignant differential diagnoses.

**Figure 3:**
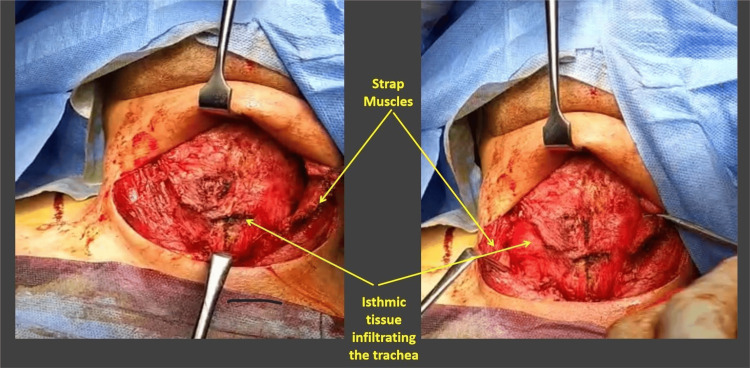
Intraoperative pictures during neck exploration 1. The thyroid gland was markedly enlarged and exhibited a rock-hard consistency. 2. Both thyroid lobes and the isthmus demonstrated extensive infiltration into the hyoid bone, cricoid cartilage, trachea, both carotid sheaths, and the surrounding soft tissues. 3. The strap muscles were completely replaced by dense fibrotic tissue.

On neck exploration, it was extremely challenging to find the dissection planes, as there was diffuse infiltration of the neck fascia, strap muscles, and all the surrounding soft tissue structures. The whole thyroid gland was extremely hard, and surprisingly, it was infiltrating the hyoid bone, cricoid cartilage, trachea, both carotid sheaths, and surrounding tissues to the degree that we decided to reverse our decision and be satisfied with resecting only the isthmic area to avoid further surgical complications for the patient and to relieve some tracheal compression (Figure 3; Appendices).

**Figure 4:**
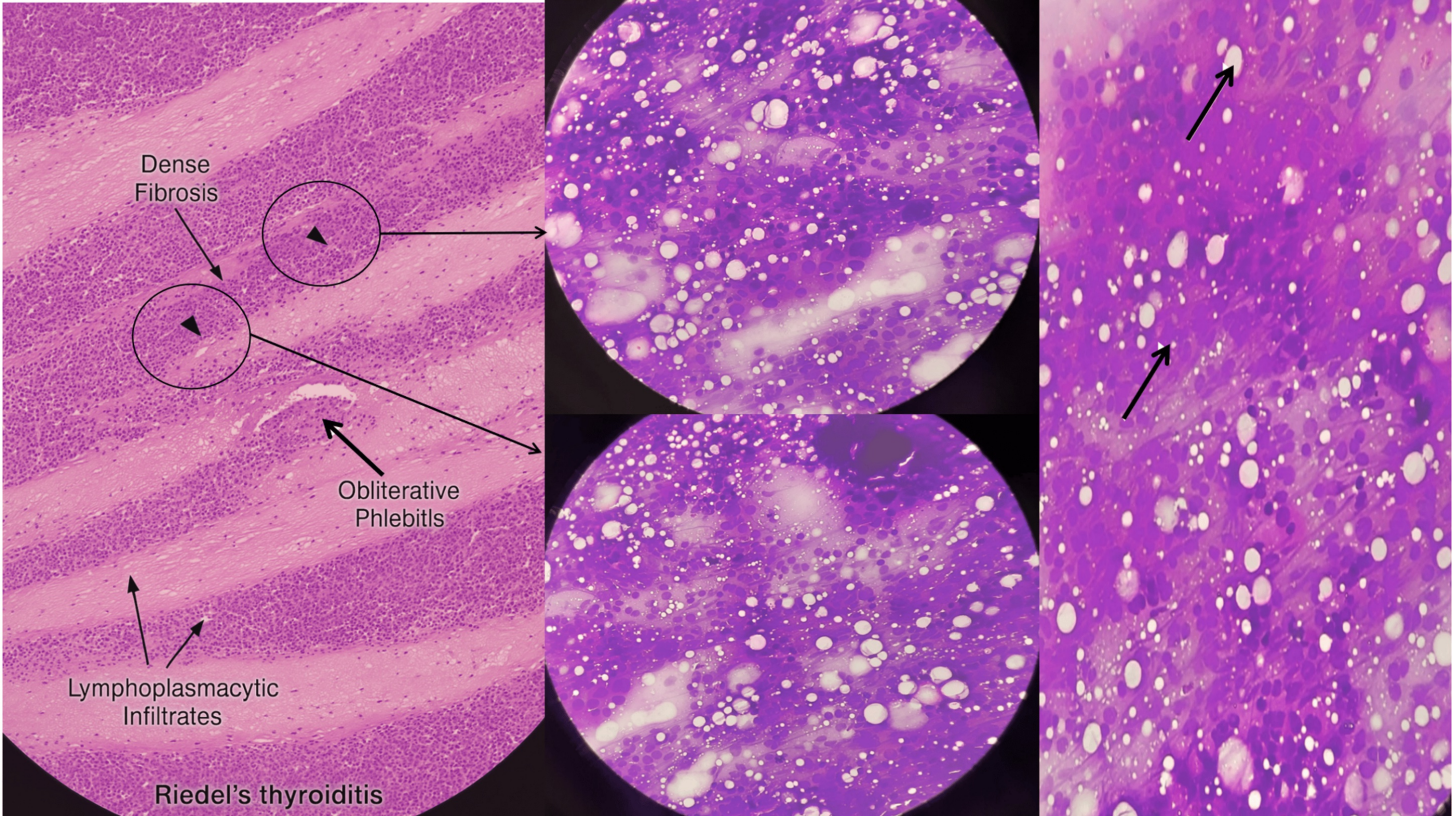
Histopathological features of Riedel’s thyroiditis (A) Low-power overview showing near-total replacement of thyroid parenchyma by dense fibrous tissue. (B) Dense fibrotic stroma entrapping residual thyroid follicles. (C) Sparse chronic inflammatory infiltrate scattered within fibrotic areas. (D) Fibrosis extending beyond the thyroid capsule into adjacent soft tissue, consistent with invasive sclerosing thyroiditis.

**Figure 5:**
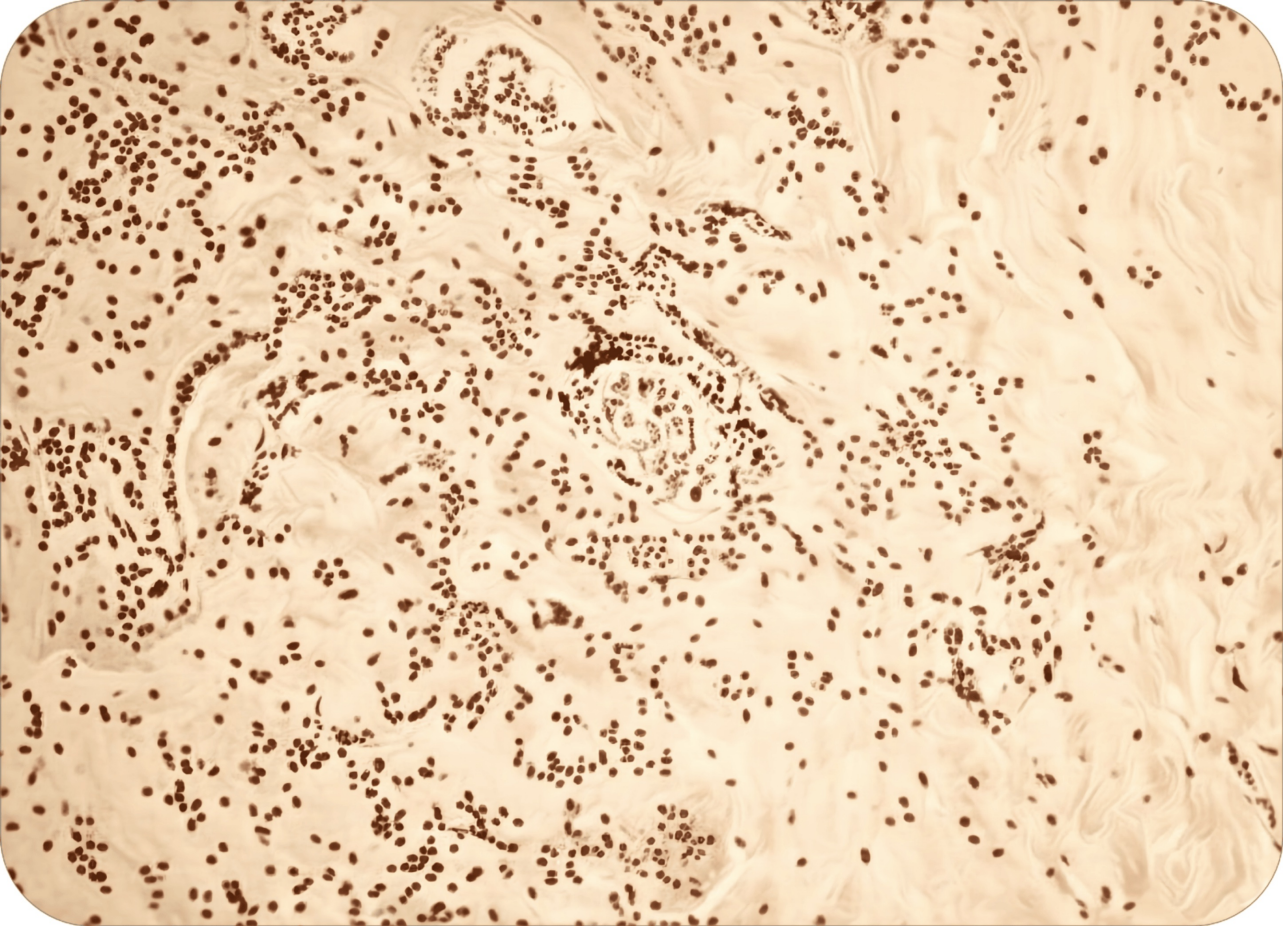
Immunohistochemistry staining (IHS) IgG4 staining highlights numerous IgG4-positive plasma cells within fibrosclerotic thyroid tissue. IgG4: immunoglobulin G subclass 4

The specimens were sent off from the removed tissue for paraffin sectioning. Final histopathology showed diffuse infiltration of abundant lymphoplasmacytic inflammatory infiltrate with some eosinophils associated with extensive fibrosis and marked blood vessel damage (Figure 4). Further immunohistochemical staining (IHS) confirmed the diagnosis, showing positive IgG4, which ruled out malignancy (Figure 5). The final diagnosis of RT was made. It was one of the rarest instances to see such an unusual presentation of a case of RT.

**Figure 6:**
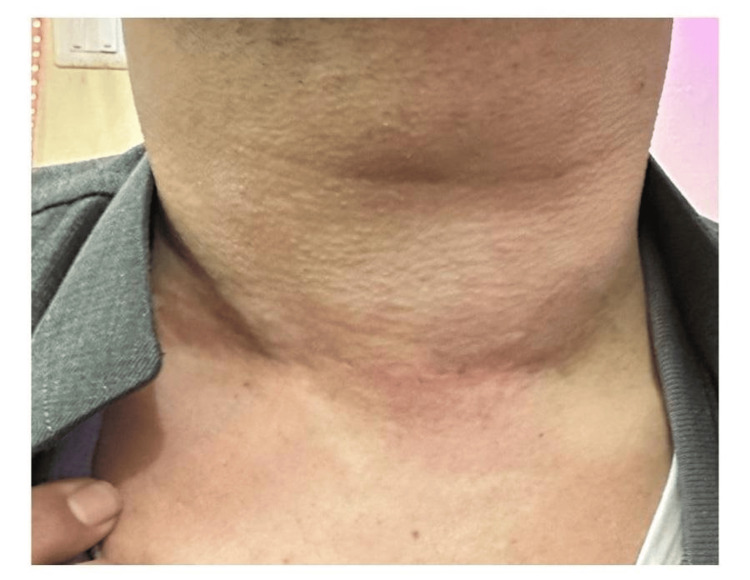
Inspection of the neck one year after surgery

The postoperative period was uneventful. The patient was then referred to the endocrinology clinic to receive medical treatment and follow-up for such a rare case. Postoperatively, L-thyroxine therapy was maintained and closely titrated according to thyroid function tests. The patient continued on L-thyroxine 150 µg/day, with TFT reassessment at 6 weeks and 12 weeks showing values within the euthyroid range (6 weeks: TSH 0.9 mIU/L, fT4 12.5 pmol/L, fT3 3.8 pmol/L; 12 weeks: TSH 1.2 mIU/L, fT4 13.3 pmol/L, fT3 4.0 pmol/L). The patient was subsequently started on large doses of prednisolone (60 mg/day). After two weeks of steroid treatment, the ESR was noted to have fallen to 12 mm/h. The corticosteroid tapering regimen was planned over six months in consultation with an endocrinology specialist, to be replaced later by tamoxifen 10 mg. After one year of follow-up, the case is completely resolved, and a recent thyroid ultrasound shows only 1 cm swelling on both sides (Figure 6; Table 1).

**Table 1:** Summary of key clinical, laboratory, and pathological findings CT: computed Tomography, FNAC: fine-needle aspiration cytology, IgG4: immunoglobulin G subclass 4

Parameter	Finding / Result	Interpretation / Comment
Age & Sex	47-year-old male	—
Presenting symptoms	Huge neck swelling, stridor, dysphagia, dyspnea.	Rapidly progressive compressive symptoms
Duration & progression	12 months total, rapid increase in 4 months (neck circumference 34→40 cm)	Suggestive of an aggressive process
Ultrasound findings	Diffuse marked hypoechogenicity, heterogeneous texture, ill-defined margins, posterior shadowing, reduced vascularity	Compatible with fibrosing thyroiditis
CT findings	Diffuse thyroid enlargement with tracheal compression, encasement of carotid sheaths, no invasion	Mimicking anaplastic carcinoma
FNAC result	Lymphoplasmacytic infiltrates; Bethesda III (indeterminate)	Non-diagnostic
Serum IgG4	Not measured (airway obstruction)	Limitation - urgency
Histopathology	Dense fibrosis, lymphoplasmacytic infiltrate, eosinophils, obliterative phlebitis	Riedel’s thyroiditis
IgG4 immunostaining	Numerous IgG4-positive plasma cells	Confirms IgG4-related thyroiditis
Treatment	L-thyroxine (150 µg/day), steroids (60 mg/day, tapered over 6 months), tamoxifen (10 mg)	Excellent response
Follow-up	1 year: near-complete regression; euthyroid	Stable outcome

## Discussion

RT remains one of the rarest thyroid disorders, accounting for less than 0.05% of all thyroid diseases [1]. Its clinical relevance lies not only in its rarity, but its etiology remains unclear, with hypotheses ranging from a localized autoimmune response to a manifestation within the spectrum of IgG4-related systemic disease. Moreover, the condition is often underdiagnosed because it mimics malignant thyroid conditions, particularly ATC and thyroid lymphoma [2]. Both RT and ATC present with a rapidly enlarging hard thyroid mass, compressive symptoms, such as dysphagia, dyspnea, and stridor, and radiological findings of local invasion. This striking similarity makes the preoperative distinction between the two entities extremely challenging, often leading to an initial clinical suspicion of malignancy, as occurred in our case [3].

However, there are some important distinguishing characteristics. ATC typically progresses rapidly over days to weeks, exhibiting aggressive local invasion and destructive behavior, whereas RT demonstrates a more indolent course, usually evolving over several weeks to months. RT is often associated with IgG4-related systemic disease (e.g., autoimmune pancreatitis, sclerosing cholangitis, or retroperitoneal fibrosis), whereas ATC lacks these IgG4-related manifestations. Serum thyroglobulin levels are usually non-diagnostic in both entities; they tend to be low or normal in ATC due to tumor dedifferentiation and nonspecific in RT. On ultrasound imaging, ATC typically presents as a large, heterogeneous hypoechoic mass with irregular or blurred margins, areas of necrosis, increased vascularity, and frequent extrathyroidal extension or metastatic lymphadenopathy. In contrast, RT commonly appears as diffuse marked hypoechogenicity with ill-defined margins, posterior acoustic shadowing, and reduced intrathyroidal vascularity, reflecting extensive fibrosis and chronic inflammation [4].

FNAC is frequently non-diagnostic in RT due to the paucity of follicular cells and the predominance of fibrous tissue and inflammatory infiltrates. In contrast, ATC typically shows pleomorphic malignant cells with high mitotic activity [5].

In our patient, FNAC yielded only lymphoplasmacytic infiltrates without malignant features (Bethesda III), which remained inconclusive even after repetition. In such scenarios, core needle biopsy (CNB) represents an essential next diagnostic step, particularly in fibrosing or infiltrative thyroid lesions such as Riedel’s thyroiditis. CNB provides larger, architecturally intact tissue cores, enabling detailed assessment of fibrosis patterns, obliterative phlebitis, and immunohistochemical markers, including IgG4 staining, thereby substantially improving diagnostic accuracy [6].

The main indication for CNB is a non-diagnostic or indeterminate FNAC result when the patient’s condition is stable and there is no immediate threat to the airway. By providing a more definitive histological diagnosis, CNB can often prevent unnecessary thyroidectomy and guide appropriate medical therapy. However, CNB should be performed only by experienced operators and under ultrasound guidance, given its higher risk of bleeding or hematoma formation, particularly in fibrotic or hypervascular glands [7].

In the present case, CNB was not pursued because the patient exhibited severe airway compression and progressive stridor, making any percutaneous intervention potentially hazardous and delaying definitive airway management. Therefore, surgical exploration was prioritized both to relieve tracheal obstruction and to establish a definitive diagnosis. This case underscores the importance of recognizing airway compromise as a relative contraindication to CNB and balancing diagnostic precision with patient safety in the management of large thyroid masses.

Histopathology remains the gold standard for diagnosis, revealing dense fibrosis replacing thyroid parenchyma, extension into surrounding soft tissues, and obliterative phlebitis. Increasingly, IHS demonstrating IgG4-positive plasma cells has been recognized as a hallmark feature of RT, supporting the hypothesis that it is part of the spectrum of IgG4-related systemic disease [6]. In our case, immunohistochemistry confirmed IgG4 positivity, reinforcing the diagnosis and excluding ATC. Serum IgG4 concentration was not obtained prior to therapy due to the urgent airway indication, which represents a limitation of our study. Future cases should aim to collect baseline and follow-up serum IgG4 levels to complement tissue diagnosis.

Management strategies differ significantly between RT and ATC. While ATC requires aggressive multimodal treatment, including surgery, radiotherapy, and chemotherapy, with poor survival outcomes, RT can often be managed with corticosteroids and tamoxifen. Steroids reduce inflammation and fibrosis, while tamoxifen exerts antifibrotic effects through the modulation of transforming growth factor-beta [8]. Our patient responded dramatically to this regimen, with almost complete regression of the thyroid mass within one year. This favorable outcome further emphasizes the importance of establishing the correct diagnosis to avoid unnecessary extensive surgery and oncological treatment.

Several studies have documented similar diagnostic dilemmas, underscoring the need for heightened awareness among clinicians [9]. Shahi N et al. and Shafi A et al. reported comparable cases in which RT closely simulated ATC clinically and radiologically, but histopathology redirected the diagnosis. In addition, the recognition of RT as part of IgG4-related disease highlights the need for systemic evaluation, as patients may also have retroperitoneal fibrosis, sclerosing cholangitis, or mediastinal fibrosis [1,2]. Thus, a multidisciplinary approach involving endocrinologists, immunologists, surgeons, pathologists, and radiologists is essential.

## Conclusions

This case highlights the diagnostic challenge and clinical importance of diagnosing Riedel’s thyroiditis (RT), a rare entity that can closely mimic anaplastic thyroid carcinoma (ATC) in both presentation and imaging. While surgery was necessary for this patient due to airway compromise, repeated fine needle aspiration cytology (FNAC) or core needle biopsy (CNB) could serve as a valuable, less invasive diagnostic pathway for patients who are hemodynamically and respiratorily stable and avoid unnecessary thyroidectomy. The dramatic response to corticosteroid and tamoxifen therapy in our patient highlights the importance of conservative management once the diagnosis is established. Clinicians should take into consideration RT in the differential diagnosis of a rapidly enlarging, hard thyroid mass to avoid unnecessary radical procedures. Ultimately, a multidisciplinary approach involving endocrinology, pathology, radiology, and surgery remains central to achieving optimal outcomes and minimizing any surgical morbidity.
